# On achieving gender equity within the liver transplantation medical and surgical workforce

**DOI:** 10.3389/frtra.2024.1396631

**Published:** 2024-08-21

**Authors:** Deborah Verran

**Affiliations:** Surgical Services, Ramsay Healthcare, Sydney, NSW, Australia

**Keywords:** liver transplantation, workforce, gender, equity, surgery, hepatology

## Abstract

Until relatively recently there has been a paucity of readily available information pertaining to the demographics of the medical and surgical workforces for the subspecialty of liver transplantation. This is relevant as it relates to whether gender equity is now being achieved across this particular workforce. This manuscript focuses on what eventually led to the recognition that more comprehensive data were required along with what is now actually known with respect to the gender ratios of the liver transplant workforce along with their related academic activities. Potential solutions to address any ongoing imbalances are also examined. The extent and range of gender disparities previously reported for other cohorts of physicians and surgeons, are also apparent amongst the liver transplant workforce in most regions of the world. This also pertains to the higher leadership positions within liver transplant centers as well as for the related editorial and scientific congress roles. Common themes/recommendations are now emerging as to how best to address the lack of progress towards gender equity. These include the development and implementation of policies, the removal of barriers to career progression, and proper governance. Ongoing actions are going to be required to achieve gender equity across the workforce in liver transplantation around the world.

## 1 Introduction

Over the last decade there have been an ever-increasing number of published manuscripts appearing in scientific journals where gender equity is the predominant focus for certain aspects of solid organ transplantation. This focus can either be on the gender composition of the relevant medical and surgical workforce (1), or on the gender ratios of the patients who are being assessed as potential candidates and/or are undergoing solid organ transplantation. These may initially appear to be two completely unrelated topics although it is becoming increasingly apparent that the former may influence the latter (2). This perspective will focus on the former as it pertains to the subspecialty of liver transplantation. This includes providing an outline of why there was an increasing focus on gender equity including within this particular subspecialty, along with what are now felt to be some of the possible solutions for addressing the various ongoing disparities.

## 2 Background

The number of females graduating from medical schools over the last 20–30 years is now close to 50% in many regions around the world. Hence it was anticipated that this would have already translated into an increasing number of female doctors attaining the full range of higher academic and or leadership positions across all of the relevant subspecialties (1–3). However, it has become increasingly apparent particularly over the last decade or so that despite these increasing numbers of female medical graduates that this has not translated into the same proportion of females being employed across all of the medical and surgical subspecialties nor were they making it into the higher leadership positions for a number of the relevant academic endeavours’ (4) (Table 1).

**Table 1:** Female participation in the workforce and higher academic activities.

Area of focus	What is known	References
Workforce	Close to 50% medical school graduates are female	(3)
	Fewer females than males in academia	(3, 4)
	Fewer females in a number of surgical subspecialties	(5, 6)
Higher academic leadership	Close to 38% of US academics are female	(3)
	Fewer female full professors (21%) and medical school Deans (16%)	(3, 6)
	Fewer females in clinical leadership positions	
Academic publishing	Females less likely to be first or senior authors of manuscripts	(4, 7)
	Females less likely to be on editorial boards of high impact journals	(8–11)
	Fewer females in Chief editors’ positions	
Involvement at Conferences	Fewer female speakers at conferences	(12, 13)
	Proportion of females on conference planning committees associated with the number of female speakers.	(12, 13)
	All male panels still a regular occurrence in some sectors	

It has also become apparent that despite there being more females numerically wise in academic medical positions in the United States (US), proportionally fewer females were ending up in the higher leadership academic type positions. A systematic review of the then available published literature from the US, Canada and the United Kingdom also revealed that organizational type barriers were being increasingly identified as contributing to barriers to the career progression of female surgeons (14). The barriers identified included—an organizational type of culture which was a hindrance to the females, as well as work/family conflict affecting the females more than the males. Some of these barriers were also being mentioned as occurring in other regions of the world as well (15).

By 2018 although there had been a steady increase in the overall numbers of females in surgical disciplines within the US, the numbers were not uniform across all of the subspecialties (5). The lowest percentage of females were found to be in Cardiothoracic surgery (16%) whilst the highest percentage were in Obstetrics and Gynecology (63.3%). Similar discrepancies were also revealed in data from Spain where there were no females employed as Cardiothoracic surgeons in the healthcare system, whereas for Ophthalmology there was close to gender parity. In addition, the males were still far more likely to be in leadership positions across all of the surgical and medical specialties by a factor of just over 3 to 1 (6). When academic related activities were further looked into including for example the authorship of manuscripts published in prominent US medical journals (4), females were less likely to be either first or senior authors for a range of medical journals and were also less likely to be invited to submit guest editorials (4). Similar results were reported for a Brazilian surgical journal (7), where 25% of the first authors were female compared to 21.8% of the last authors being female. Closer examination of the membership of the editorial boards of surgical journals in the US, Europe, and Latin America as well as medical and surgical journals in Australasia (8–11), also revealed similar discrepancies. The proportion of editorial board members who were female ranged from 13 to 33.9%, whilst in comparison 4.8%–22% of the Chief Editors were female (8–11). Plus, a number of journals were identified where there were either no females on the editorial board at all, or there were no females in either an Associate or Chief editors’ position, confirming that there is significant variation in the gender ratios for who is being recruited to serve in all of these positions. Similar demographic data have been published for surgical conferences where the percentages of female speakers varied from 18.9% to 58.5%, depending on the actual subspecialty which correlated with whether or not there were females on the planning committee (12). An even larger data set for the proportion of female speakers for 98 medical meetings involving 20 specialties between 2017 and 2018 revealed that 30% of the speakers were female but that concerningly 36.6% of the panels were all male (13). Again, there was a positive correlation demonstrated between the proportion of women on the planning committee and the numbers of female speakers.

## 3 Gender ratios of the liver transplant workforce

A small number of publications have provided some idea as to what may be the current situation in recent times within a couple of countries. In Spain although 56.3% of the Hepatology workforce are female, only 15% of the high-ranking leadership type positions are held by females (3, 16). In comparison in the United States in the parent specialty of gastroenterology, although there are proportionally fewer females (15%), they do tend to be more likely to undertake an academic career (40%), but are also less likely to be found in leadership positions either within the institutions where they are employed or at the level of national societies (4, 17). The American Society of Transplant Surgeons (ASTS) has undertaken a number of surveys of its workforce over the years, such that by 2015 18% of the transplant surgeons in the United States were female, although it was not known how many of these were actually in the liver transplant subspecialty (18).

When it comes to other academic type activities such as publishing in the hepatology related scientific journals data have been published for the genders of the first and second authors of published manuscripts as well as for the invited editorials/reviewers of liver related publications (15, 19). It appears that female authors regardless of their career stage are less well represented (ranging from 20.3% of the senior authors being female through to 34.9% of the first authors being female). In fact, there was some evidence that the percentage of female senior authors diminished over a 2-year period (2014–2015) from 32.6% to 20.3%. Data has also been published on the ratios of females to males for both the chief editors’ positions (7.7% were female), along with the wider editorial boards (17.4% were female) of some of the high impact gastroenterology and hepatology journals (5, 20). There are also published data highlighting the discrepancies in the gender composition of various chief editors’ positions (4%–17% were female), for a range of solid organ transplantation journals (6, 21). As to how many of these females were surgeons was not able to be ascertained from any of these publications.

It also appears that proportionately fewer female attendees (19%) deliver presentations at hepatology related professional scientific meetings, which mirrors the proportion of both the session convenors and panel moderators who are female (20%) (2). Whereas in Spain where on average 60% of the presenters of abstracts at the relevant scientific meetings are female, in comparison only 19% of the invited speakers or the session moderators are female (3, 16). When it comes to the involvement of female liver transplant surgeons in scientific meetings even less is known. Examination of the programs for the American Society of Transplant Surgeons (ASTS) annual Winter Symposium meeting between 2015 and 2019 have revealed that female participation has improved in recent years (22). This includes there being an increase in the number of females speakers from 20% to 40% as well as an increase in the number of female panellists from 0% to 30%, with a commensurate decrease in the number of all male panels. However, it was not known how many of these females were liver transplant surgeons. Mention has been made of this gender disparity also extending to the awarding of research grants (7, 23), including from major research funders within the United States. However currently there is a lack of published granular data pertaining to the awarding of research grants in other regions of the world particularly when it comes to research in the various liver transplantation related disciplines. Hence it seems on balance that there may be barriers to the females also securing the types of other senior roles and or related positions which are associated with both career advancement as well as attaining the higher levels of academia in Hepatology as well as in liver transplant surgery. This is commonly known as the pipeline effect (8, 9, 24, 25), where women are lost from every level of the academic career ladder.

As to understanding what might also be happening internationally when it comes to the gender mix of the liver transplant medical and surgical workforce up until, relatively recently there was a relative paucity of data. A clearer picture began to emerge when the results of data collected from 243 transplant centres were published via a subcommittee of the International Liver Transplantation Society (ILTS) (14, 26). This revealed that only 32/243 centres (13.2%) had either a female chief of Hepatology or of Liver Transplant Surgery. In addition, 31.9% of the responding hepatologists were female compared to 18.2% of the transplant surgeons (14). However, the percentages of females who were in a leadership position varied according to practice location, with there being no females in leadership positions in either Australia or Canada whilst the highest proportion were based in the United States (18%). This is similar to 17% of the Chiefs of Transplant Surgery across the United States being female (27), although this percentage may not be the same for the heads of the clinical liver transplant programmes. The ILTS survey also revealed that the percentage of liver transplant programs without either a female hepatologist or a female liver transplant surgeon on staff, also varied from region to region with the lowest rates seen in Asia (57% and 77% respectively). However, the percentage of the total number of female members of the ILTS (26.4%), was similar to the percentage of females who were holding leadership positions within the ILTS (26.9%), which the authors partly attributed to ongoing efforts to promote females into leadership positions within the society (26). To date this is the most comprehensive set of data that has become available despite the fact that not all of the transplant centres were captured and not every liver transplant professional is a member of the ILTS. Mention has been made of more comprehensive data capture methods and reporting being used by the relevant organizations in order to better understand what the true extent of the various gender disparities may be (26).

## 4 Recommendations for addressing the gender disparities

It is apparent that a number of generic type of recommendations for corrective measures have steadily emerged via a number of the previously mentioned publications (Table 2), many of which pertain to all of the related stakeholder organizations in healthcare. The first step involves recognising that there may be an ongoing gender disparity of the relevant workforce within the organization and that this disparity may be more marked for the higher leadership positions. The next step involves developing and implementing policies that encompass a range of corrective actions, including both sponsoring female professionals as well as removing barriers to career progression. This may also require implementation of specific context relevant policies for example around both remuneration and family leave. A lack of adequate family leave entitlements has been previously mentioned as a barrier to career progression for females (14, 26, 28). This may be particularly relevant for the female liver transplant surgeons, noting the data pertaining to the heavy call schedules and long weekly working hours for transplant surgeons practising in the United States (18). These types of measures also need to be implemented across the wider healthcare organizations as the relevant issues are generic and hence also apply to the rest of the female medical and surgical workforce. Plus, it is increasingly being recognised that isolated stand-alone sponsorship and/or mentorship initiatives on their own may not result in more females either being employed in the relevant subspecialties and/or attaining higher leadership positions (1, 25, 28).

**Table 2:** Recommended actions to achieve gender equity.

Actions	Relevant organizations	References
Recognize that this is an issue	Healthcare employers	(1, 14, 26, 28)
	Professional societies	
	Scientific journals Research funding organizations	
Develop and implement policies	Healthcare systems/organizations	
	Professional societies Scientific journals	(9–11, 19)
Address barriers to career progression	Within healthcare organizations	(10, 14, 26, 28)
	Within professional societies	
	Within individual scientific journals	(4, 5, 17, 19)
	Within academic organizations	
Sponsor females into leadership positions	All healthcare stakeholder groups	(1, 24, 28)
	Research/industry stakeholders	

When it comes to the relevant scientific journals, this may require a range of specific corrective actions. This includes ensuring that more females are recruited onto the wider editorial board, as well as facilitating more females attaining either the Chief editor or the Associate editor roles (9–11, 19). Attention also needs to be paid to the gender ratios of the authors for invited editorials and or perspective type manuscripts. In addition, for transparency purposes it would be useful if the relevant scientific journals could both track and publish the data on the gender ratios of both first and senior authors as well as for the invited authors.

There are also connotations for all of the relevant professional stakeholder organizations of which there are a number of these based around the world. Several of the larger international professional solid organ transplant type organizations have already undertaken to move from just having formal statements on equity and diversity to understanding what is actually happening at the level of the workforce. The ILTS and the ASTS have already been mentioned, however more recently the European Society for Organ Transplantation (ESOT) formally surveyed its membership on their views on diversity and equity. There was significant support for the society moving towards prioritizing a number of ongoing efforts to embed equity and diversity initiatives into all of its professional activities (1). This involves undertaking a number of deliberate actions to ensure that gender equity and diversity is being achieved across the range of leadership positions within ESOT. There was also support for action pertaining to achieving both equity and diversity in the awarding of research grants as well as for all other awards being sponsored by the society. Although, the data being obtained by these particular organizations via surveying their membership is proving to be useful, it does seem clear that more data may need to be collected in order to gain a greater understanding of whether progress is being made with the recruitment of as well as the career progression of their female liver transplant professionals.

All of these aforementioned measures are important because with liver transplantation being a relatively small subspecialty located within wider healthcare systems and/or organizations around the world, there also needs to be an ongoing focus on the pertinent wider system level actions. This has a number of connotations particularly for the leadership and governance of all of the types of stakeholder organizations that are mentioned in Table 2. Along with implementing a range of actions, equally important will be the capturing and reporting of the relevant data elements. This is essential so that a greater understanding can be obtained of not only where the ongoing issues might lie but also whether progress is being made in reducing the current documented gender disparities for the liver transplant workforce.

## 5 Conclusions

It is increasingly becoming apparent that both the range and extent of gender disparities that have been previously been documented to be evident amongst some other cohorts of physicians and surgeons appear to be evident amongst the relevant liver transplant workforce around the world. A number of recommendations have been proposed to address these various disparities based on what is already known as well as from surveying some of these liver transplant professionals. Although more research is required, there are already a number of significant connotations that are of relevance in particular to the professionals who are in leadership positions in the relevant stakeholder organizations.

## Data Availability

The original contributions presented in the study are included in the article/Supplementary Material, further inquiries can be directed to the corresponding author.
